# Efficacy of Pharmacological Interventions in Milder Depression: A Systematic Review and Meta‐Analysis

**DOI:** 10.1002/npr2.70008

**Published:** 2025-02-27

**Authors:** Minoru Urata, Hitoshi Sakurai, Fumihiko Ueno, Taku Maruki, Teruo Tada, Takahito Uchida, Yasuyuki Matsumoto, Masami Murao, Masayuki Tomita, Hajime Baba, Masaki Kato, Takashi Tsuboi, Koichiro Watanabe

**Affiliations:** ^1^ Department of Neuropsychiatry Kyorin University Faculty of Medicine Tokyo Japan; ^2^ Brain Health Imaging Centre Centre for Addiction and Mental Health Toronto Ontario Canada; ^3^ Uchida Clinic Tokyo Japan; ^4^ Department of Neuropsychiatry Keio University School of Medicine Tokyo Japan; ^5^ Oizumi Hospital Tokyo Japan; ^6^ Department of Psychiatry & Behavioral Science Juntendo University Graduate School of Medicine Tokyo Japan; ^7^ Department of Neuropsychiatry Kansai Medical University Osaka Japan

**Keywords:** mild depression, omega‐3 fatty acids, pharmacotherapy, St. John's Wort, supplements

## Abstract

**Background:**

Mild depression lacks a consistent definition across diagnostic criteria and rating scales, posing challenges to standardizing treatment strategies. International guidelines predominantly recommend psychotherapy as the first‐line treatment for mild depression, while the use of antidepressants remains contentious. Supplements such as omega‐3 fatty acids, St. John's Wort, and magnesium have garnered attention as alternative therapeutic options for depression. This systematic review aims to assess the efficacy of pharmacological interventions, including supplements, in the treatment of mild depression.

**Methods:**

Comprehensive searches were performed in PubMed and Embase through November 2024 to identify randomized controlled trials (RCTs) investigating pharmacotherapy or supplements for mild depression diagnosed using standardized criteria. Eligible studies underwent screening and risk of bias assessment utilizing the ROB2 tool. Data on remission rates, symptom improvement, dropout rates, and adverse events were extracted, with meta‐analyses conducted where applicable.

**Results:**

Eight RCTs comprising 1049 participants met inclusion criteria. Among the agents studied, St. John's Wort was analyzed in two trials, both comparing it to fluoxetine. A meta‐analysis found no significant difference in response rates between the two treatments (risk ratio [RR] = 0.96, 95% CI: 0.78–1.18) or dropout rates (RR = 1.08, 95% CI: 0.62–1.88). For other agents, single studies evaluated their effects. Eicosapentaenoic acid and *Rhodiola rosea* demonstrated significant improvements in depressive symptoms compared to placebo. In non‐blinded trials, magnesium chloride showed efficacy in alleviating depressive symptoms. Other interventions, such as lavender, lemon balm, and transcranial electroacupuncture stimulation, were as effective as antidepressants. Conversely, S‐adenosylmethionine did not produce significant improvements relative to placebo.

**Conclusion:**

This review demonstrates that certain supplements, such as eicosapentaenoic acids and *Rhodiola rosea* , are therapeutic options for mild depression. However, no RCTs compared antidepressants directly to placebo for mild depression. The paucity of high‐quality RCTs exclusively targeting mild depression limits definitive conclusions, warranting further rigorous research.

## Abstract 2

This systematic review examined pharmacological interventions for mild depression; however, no trials were identified that specifically compared antidepressants with placebo exclusively in patients with mild depression. Among supplements, eicosapentaenoic acid and *Rhodiola rosea* emerged as potential treatment options. The scarcity of high‐quality studies highlights the need for further rigorous research to inform evidence‐based treatment strategies.

## Graphical abstract

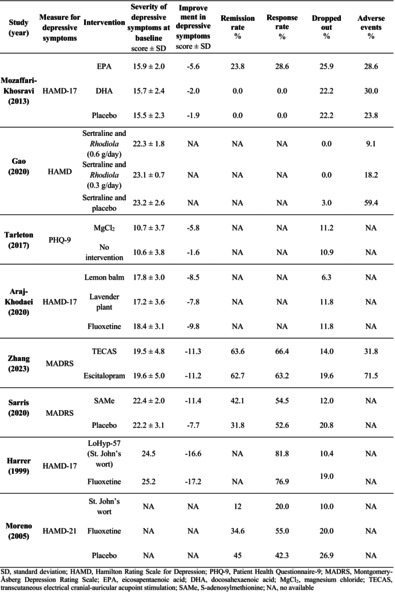

## 1 Background

Mild depression is variably defined across diagnostic criteria and rating scales. The International Classification of Diseases (ICD) and the Diagnostic and Statistical Manual of Mental Disorders (DSM) classify mild depression as the presence of a minimum number of symptoms without severe functional impairment [1, 2]. Rating scales, including the Hamilton Depression Rating Scale (HAM‐D), assess depression severity on a spectrum according to symptom scores [3]. Some clinical studies also consider cases with subthreshold depressive symptoms within the scope of mild depression [4]. A unified consensus among guidelines remains lacking, as the National Institute for Health and Care Excellence (NICE) guidelines define depression more broadly as “less severe” and “more severe.”

Most international practice guidelines recommend initiating basic psychotherapy as the primary intervention for mild depression, with varying levels of endorsement for the use of antidepressants. The Canadian Network for Mood and Anxiety Treatments (CANMAT) guidelines advocate for psychotherapy as the first‐line treatment for mild depression due to its favorable risk–benefit profile, noting that both pharmacotherapy and psychotherapy are effective [5]. The NICE guidelines prioritize guided self‐help as a minimally invasive, resource‐efficient intervention for mild cases, while antidepressants are generally not recommended as a first‐line treatment unless specifically requested by the patient [6]. Despite these recommendations, psychotherapy has notable limitations, including significant time requirements and the need for therapists with extensive training.

Recent meta‐analyses of individual patient data have produced mixed results regarding the efficacy of antidepressants in mild depression. A meta‐analysis of six placebo‐controlled randomized controlled trials (RCTs) found no significant interaction between baseline depression severity and treatment outcomes for newer antidepressants [7]. Likewise, an analysis of 13 placebo‐controlled RCTs for duloxetine showed symptom improvement irrespective of baseline severity [8]. In contrast, a meta‐analysis of 28 studies with 8262 participants reported a positive correlation between baseline depression severity and the efficacy of selective serotonin reuptake inhibitors (SSRIs) [9]. Another analysis of 236 placebo‐controlled RCTs found that the efficacy of antidepressants over placebo increased significantly with higher baseline depression severity [10]. While some studies suggest pharmacotherapy may be less effective for mild depression, others indicate equal effectiveness across all levels of severity, leaving a clear conclusion uncertain.

Additionally, the use of supplements as alternative treatments for depression has garnered renewed attention. According to the CANMAT guidelines, St. John's Wort is recommended as a first‐line treatment for mild depression among herbal remedies, with saffron, lavender, and Rhodiola listed as third‐line options [5]. Naturally occurring substances such as S‐adenosyl‐L‐methionine (SAM‐e) and omega‐3 fatty acids are also categorized as third‐line [5]. Although the recommendation of these supplements remains tentative due to the limited strength of supporting evidence, they are still acknowledged as potential therapeutic options. Further research into their efficacy for treating mild depression is encouraged.

Several systematic reviews have assessed the efficacy of pharmacological interventions for low‐severity depression, including subthreshold cases; however, these reviews did not exclusively focus on a confirmed diagnosis of depression [11]. We therefore systematically reviewed the efficacy of pharmacological interventions, including supplements, specifically for patients diagnosed with mild depression according to diagnostic criteria and conducted a meta‐analysis of the results where feasible.

## 2 Methods

This study was conducted in accordance with the PRISMA recommendations for reporting systematic reviews and meta‐analyses [12] and was registered on the Open Science Framework (https://osf.io/7a3fe).

### 2.1 Search Strategy

We searched the electronic databases, PubMed (search date: November 26, 2024) and Embase (search date: November 26, 2024), for RCTs, using appropriate subject headings and relevant search terms (e.g., “mild” and “antidepressant*” see Table S1). When necessary, we contacted the authors of specific studies to clarify additional points.

### 2.2 Inclusion and Exclusion Criteria

Studies meeting the following criteria were included in the review:

1. RCTs that investigate the efficacy of antidepressants, supplements, or analogous interventions.
2. Individuals diagnosed with major depressive disorder are based on operational diagnostic criteria.
3. Studies originally focused on either mild or mild to moderate depression, as determined by assessment scales.
4. Papers composed in the English language.

Studies meeting the following criteria were excluded from the review:

1. Studies involving depression with concurrent physical complications.
2. Research encompassing persistent depressive disorders or subthreshold depression.

The criteria for defining mild depression were established as follows: the HAMD‐17: 8–13, the Montgomery‐Åsberg Depression Rating Scale (MADRS): 7–19, the Patient Health Questionnaire‐9 (PHQ‐9): 5–9, the Beck Depression Inventory‐II (BDI‐II): 14–19, the Geriatric Depression Scale‐15: 5–8, the Edinburgh Postnatal Depression Scale: 9–11, and the Self Rating Depression Scale: 51–59. While the delineation of mild depression based on the HAMD‐17 score exhibits variability across literature, the present study adheres to the definition provided by the American Psychiatric Association guidelines [13]. Other assessment scales generally present consistent perspectives on the mild range, with its classification determined based on original sources and prior research [14, 15, 16, 17, 18, 19].

### 2.3 Article Selection Process

The titles and abstracts of identified references underwent independent screening by two authors (MU and FU), and irrelevant studies were excluded. Subsequently, the full texts of these references were independently evaluated by the two authors, and reports deemed ineligible were excluded based on the aforementioned criteria. Any discrepancies in opinions between the screening reviewers were resolved by another author (HS) through comprehensive and systematic discussion. Following the identification of eligible studies, the full texts of each study were examined.

### 2.4 Data Extraction

The subsequent data were extracted from the included studies: sample size; demographic characteristics of the study population; treatment setting; duration of the study; initial depression scores; changes in depression scores; remission rates; response rates; dropout rates; and incidence of adverse events. Any absent data were sought from the authors. In cases where the HAMD was employed without specification of its version, it was presumed that the original HAMD‐17 was utilized.

### 2.5 Assessment of Risk of Bias

The quality of the included studies was evaluated using a revised Cochrane risk of bias tool for randomized trials (ROB2) by one author (MU) and a discrepancy check was then performed by another author (HS). Items assessed included (1) bias arising from the randomization process, (2) bias due to deviations from the intended interventions, (3) bias due to missing outcome data, (4) bias in the measurement of the outcome, and (5) bias in the selection of the reported result. The quality of the studies included in each domain was assessed using three options: “low risk,” “some concerns” and “high risk.” The risk‐of‐bias visualization (robvis) tool was used to make figures that summarize the risk‐of‐bias assessments [20].

### 2.6 Statistical Analyses

The Cochrane Collaboration Review Manager software (RevMan 5.4.1) was used for statistical analysis. Dichotomous variables (remission rate and dropout rate) were measured by risk ratio (RR). The corresponding 95% confidence interval (CI) was calculated. Random effects models were used in the analyses.

## 3 Results

### 3.1 Description of Studies Included in the Review

**FIGURE 1:**
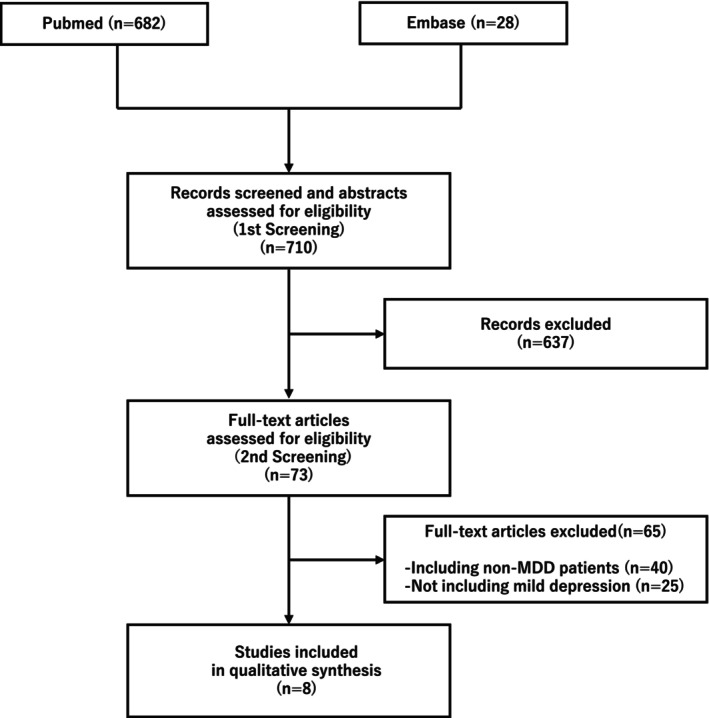
Flowchart. MDD, major depressive disorder.

The initial literature search yielded 710 unique entries published until November 2024 (PubMed, 682; Embase, 28). After carefully reviewing the titles and abstracts of the identified articles, the full‐text versions of 73 articles underwent examination. Subsequently, 65 articles were excluded following a thorough appraisal of the full text, while 8 articles met the inclusion criteria and were retained for further analysis (Figure 1) [21, 22, 23, 24, 25, 26, 27, 28].

### 3.2 Study Characteristics

Eight articles published between 1999 and 2023 were included in this review. The sample size ranged from 45 to 468, with a total of 1049 participants (Table 1) [21, 22, 23, 24, 25, 26, 27, 28]. Among the participants, 71.0% were female, and the mean age was 45.1 years. The duration of these studies spanned from 6 to 12 weeks. The criteria used for the diagnosis of major depressive disorder varied across studies. Three studies utilized the DSM‐IV [21, 22, 28]. Two studies adhered to the DSM‐5 [24, 26]. Two studies followed ICD‐10 [25, 27]. Another study included patients with a documented diagnosis of depression in their medical records [23]. Six studies employed a double‐blind design, ensuring both participants and assessors were blinded to the treatment allocation [21, 22, 24, 26, 27, 28]. One study utilized an assessor‐blind approach [25], while another was conducted as an open‐label trial [23].

**TABLE 1:** Characteristics of study participants.

Study (year)	Intervention	Age years ± SD	Female *n* (%)	Diagnosis	Severity	Study duration weeks	Setting
Mozaffari‐Khosravi (2013)	EPA	37.5 ± 12.4	13/21 (61.9)	Mild‐to‐moderate depression (DSM‐IV)	HAMD‐17: 8–18	12	Outpatients in Iran
	DHA	34.0 ± 13.1	12/20 (60.0)				
	Placebo	33.8 ± 9.9	13/21 (61.9)				
Gao (2020)	Sertraline and *Rhodiola* (0.6 g/day)	38 ± 10.6	19/33 (57.6)	Major depressive disorder (DSM‐IV)	HAMD≧ 12, excluded severe MDD and those with CGI: 3–4	12	Patients in China
	Sertraline and *Rhodiola* (0.3 g/day)	39 ± 12.9	17/33 (51.5)				
	Sertraline and placebo	35 ± 13.2	17/32 (53.1)				
Tarleton (2017)	MgCl_2_	55.2 ± 12.3	33/55 (60.0)	Diagnosis of depression in their medical record	PHQ‐9: 5–19	6	Outpatients in the US
	No intervention	50.1 ± 13.0	35/57 (61.4)				
Araj‐Khodaei (2020)	Lemon balm	37.4 ± 3.3	10/15 (66.7)	Mild‐to‐moderate depression (DSM‐5)	HAMD‐17: 8–24	8	Outpatients in Iran
	Lavender plant	37.9 ± 2.4	11/15 (73.3)				
	Fluoxetine	33.4 ± 2.7	11/15 (73.3)				
Zhang (2023)	TECAS	39.9 ± 13.7	163/233 (70.0)	Major depressive episode (ICD‐10)	MADRS: 12–29	8	Outpatients in China
	Escitalopram	40.6 ± 12.9	170/235 (72.3)				
Sarris (2020)	SAMe	45.2 ± 12.6	18/25 (72.0)	Major depressive disorder (DSM‐5)	MADRS: 14–25	8	Patients in Australia
	Placebo	42.0 ± 12.3	21/24 (87/5)				
Harrer (1999)	LoHyp‐57 (St. John's Wort)	68.4	60/70 (85.7)	Mild‐to‐moderate depressive episode (ICD‐10)	SDS: 51–59	6	Outpatients in Germany
	Fluoxetine	69.1	69/79 (87.3)				
Moreno (2005)	St. John's Wort	37.2 ± 11.7	44/53 (83.0) [Total for all 3 groups]	Major depressive disorder (DSM‐IV)	HAMD‐21: 10–24	8	Outpatients in Brazil
	Fluoxetine	37.7 ± 9.9					
	Placebo	45.9 ± 10.8					

Abbreviations: DHA, docosahexaenoic acid; DSM, Diagnostic and Statistical Manual of Mental Disorders; EPA, eicosapentaenoic acid; HAMD, Hamilton Rating Scale for Depression; ICD, International Classification of Diseases; MADRS, Montgomery‐Åsberg Depression Rating Scale; MDD, major depressive disorder; MgCl_2_, magnesium chloride; PHQ‐9, Patient Health Questionnaire‐9; SAMe, S‐adenosylmethionine; SD, standard deviation; SDS, Self Rating Depression Scale; TECAS, transcutaneous electrical cranial‐auricular acupoint stimulation.

### 3.3 Risk of Bias Assessment

**FIGURE 2:**
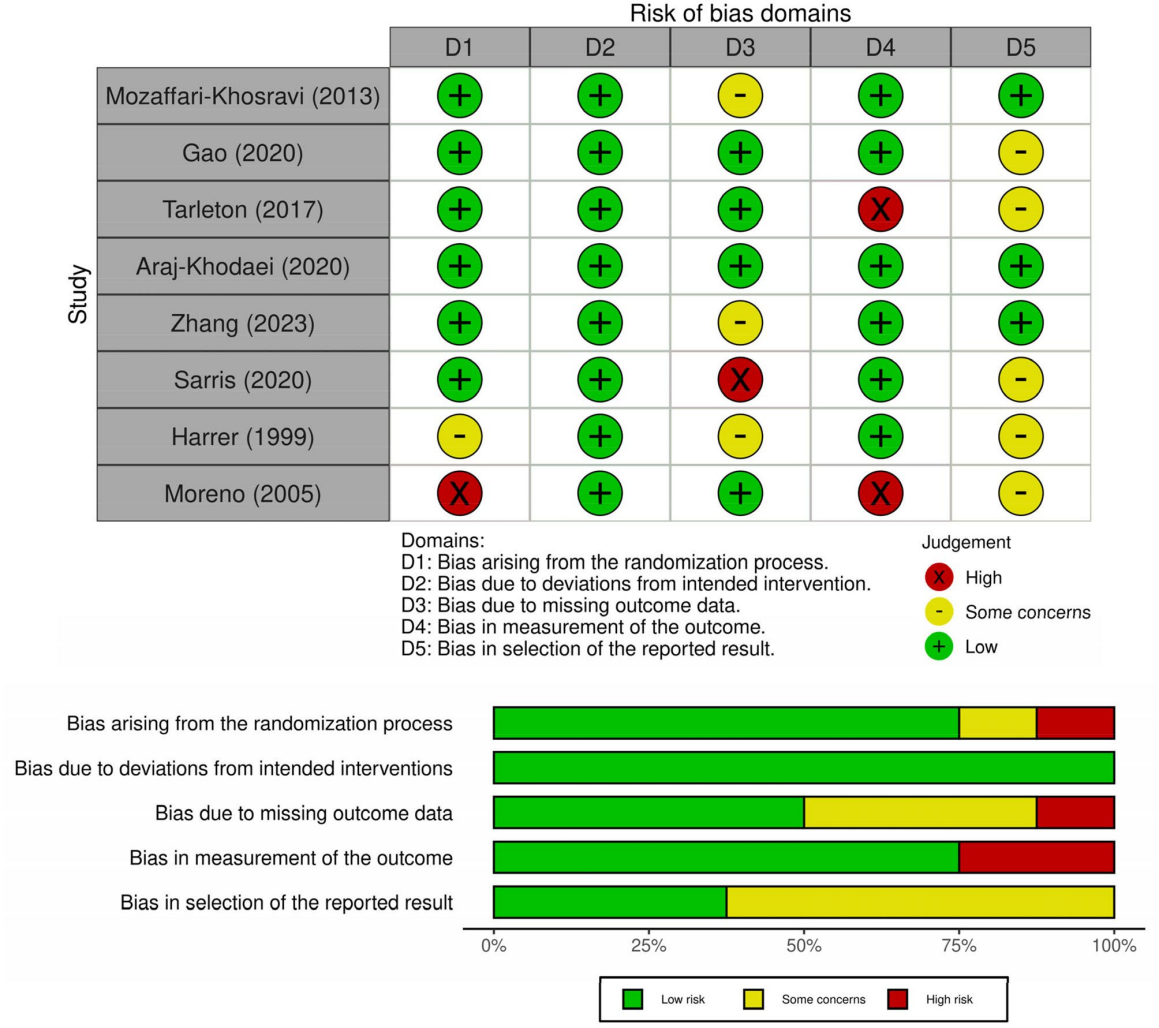
Risk of bias assessment. Green indicates a low risk of bias, yellow indicates some concerns, and red indicates a high risk of bias.

The risk of bias assessment (Figure 2) yielded the following findings: six RCTs utilized appropriate randomization methods and outcome measurement; all RCTs demonstrated a low risk of bias in the domain concerning deviations from intended interventions; however, one RCT was deemed to have a high risk of bias due to missing outcome data. Regarding the selection of the reported results, five RCTs presented some concerns of bias due to uncertainty about whether the analysis adhered to the research plan.

### 3.4 Treatment Outcome Assessment

The outcomes are summarized in Table 2. In a 12‐week double‐blind RCT involving 81 outpatients diagnosed with milder depression, eicosapentaenoic acid (EPA), typically prescribed for hyperlipidemia, demonstrated a significant improvement in the HAMD‐17 scores compared to docosahexaenoic acid (DHA) and placebo [21]. In another double‐blind trial comparing an extract from *Rhodiola rosea* roots, included in official Russian medicine, added to sertraline versus placebo added to sertraline in 100 patients with milder depression, *Rhodiola rosea* significantly improved the HAMD‐17 scores after 12 weeks [22]. A non‐blinded trial comparing magnesium chloride to no treatment in 126 outpatients with milder depression demonstrated a significant improvement in the PHQ‐9 scores after 6 weeks with magnesium chloride [23]. In an 8‐week double‐blind trial involving 45 outpatients with milder depression, lemon balm, lavender, and fluoxetine showed no significant difference in the HAMD‐17 score changes among the three interventions [24]. An assessor‐blind trial involving 468 patients with milder depression found no significant difference in treatment response rates after 8 weeks between transcutaneous electrical cranial–auricular acupoint stimulation and escitalopram [25]. A double‐blind trial comparing S‐adenosylmethionine (SAMe) to placebo in 49 patients with milder depression showed no significant difference in the MADRS score changes after 8 weeks [26]. A double‐blind trial comparing St. John's Wort dry extract to fluoxetine in 149 patients with milder depression found no significant difference in the HAMD‐17 score changes after 8 weeks [27]. On the other hand, in a double‐blind trial comparing St. John's Wort dry extract, fluoxetine, and placebo in 66 outpatients with milder depression, St. John's Wort showed significantly lower response and remission rates based on the HAMD‐21 scores at 8 weeks compared to fluoxetine and placebo [28].

**TABLE 2:** Summary of outcomes.

Study (year)	Measure for depressive symptoms	Intervention	Severity of depressive symptoms at baseline score ± SD	Improvement in depressive symptoms score ± SD	Remission rate %	Response rate %	Dropped out %	Adverse events %
Mozaffari‐Khosravi (2013)	HAMD‐17	EPA	15.9 ± 2.0	−5.6	23.8	28.6	25.9	28.6
		DHA	15.7 ± 2.4	−2.0	0.0	0.0	22.2	30.0
		Placebo	15.5 ± 2.3	−1.9	0.0	0.0	22.2	23.8
Gao (2020)	HAMD	Sertraline and *Rhodiola* (0.6 g/day)	22.3 ± 1.8	NA	NA	NA	0.0	9.1
		Sertraline and *Rhodiola* (0.3 g/day)	23.1 ± 0.7	NA	NA	NA	0.0	18.2
		Sertraline and placebo	23.2 ± 2.6	NA	NA	NA	3.0	59.4
Tarleton (2017)	PHQ‐9	MgCl_2_	10.7 ± 3.7	−5.8	NA	NA	11.2	NA
		No intervention	10.6 ± 3.8	−1.6	NA	NA	10.9	NA
Araj‐Khodaei (2020)	HAMD‐17	Lemon balm	17.8 ± 3.0	−8.5	NA	NA	6.3	NA
		Lavender plant	17.2 ± 3.6	−7.8	NA	NA	11.8	NA
		Fluoxetine	18.4 ± 3.1	−9.8	NA	NA	11.8	NA
Zhang (2023)	MADRS	TECAS	19.5 ± 4.8	−11.3	63.6	66.4	14.0	31.8
		Escitalopram	19.6 ± 5.0	−11.2	62.7	63.2	19.6	71.5
Sarris (2020)	MADRS	SAMe	22.4 ± 2.0	−11.4	42.1	54.5	12.0	NA
		Placebo	22.2 ± 3.1	−7.7	31.8	52.6	20.8	NA
Harrer (1999)	HAMD‐17	LoHyp‐57 (St. John's Wort)	24.5	−16.6	NA	81.8	10.4	NA
		Fluoxetine	25.2	−17.2	NA	76.9	19.0	NA
Moreno (2005)	HAMD‐21	St. John's Wort	NA	NA	12	20.0	10.0	NA
		Fluoxetine	NA	NA	34.6	55.0	20.0	NA
		Placebo	NA	NA	45	42.3	26.9	NA

Abbreviations: DHA, docosahexaenoic acid; EPA, eicosapentaenoic acid; HAMD, Hamilton Rating Scale for Depression; MADRS, Montgomery‐Åsberg Depression Rating Scale; MgCl_2_, magnesium chloride; NA, no available; PHQ‐9, Patient Health Questionnaire‐9; SAMe, S‐adenosylmethionine; SD, standard deviation; TECAS, transcutaneous electrical cranial‐auricular acupoint stimulation.

### 3.5 Meta‐Analysis

**FIGURE 3:**
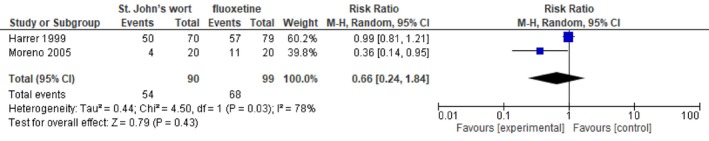
Forest plot of post‐intervention treatment effect sizes for remission rate from depressive symptoms. CI, confidence interval.

**FIGURE 4:**
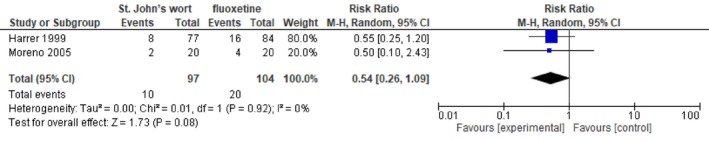
Forest plot of post‐intervention treatment effect sizes for dropout rate for all reasons during study period. CI, confidence interval.

Two articles comparing the effects of St. John's Wort and fluoxetine were identified [27, 28]. The results from these studies were combined to evaluate the efficacy of St. John's Wort relative to fluoxetine. The analysis revealed no significant difference between the St. John's Wort and fluoxetine groups in the response rates (RR: 0.66, 95% CI: 0.24–1.84, *p* = 0.43) (Figure 3) and dropout rates for all reasons during the study period (RR: 0.54, 95% CI: 0.26–1.09, *p* = 0.08) (Figure 4).

## 4 Discussion

This study is the first systematic review focused on pharmacological interventions specifically for milder cases of major depressive disorder. In the systematic search conducted for this review, no RCTs were found that evaluated the efficacy of antidepressants exclusively in cases of mild depression. On the other hand, several RCTs were identified that targeted patients with relatively mild depression and examined the use of supplements, which could represent viable treatment options for mild depression.

Omega‐3 fatty acids, commonly used as lipid‐lowering agents and available from dietary sources such as fatty fish, have demonstrated potential therapeutic effects in depression. A meta‐analysis of 14 studies investigating plasma omega‐3 fatty acid levels in patients with depression showed significantly lower levels in depressed individuals compared to controls [29]. Postmortem studies have also reported notably lower concentrations of omega‐3 fatty acids in the prefrontal cortex of individuals with depression [30]. Additionally, lower omega‐3 levels have been linked to poor responsiveness to antidepressants, suggesting a connection between omega‐3 deficiency and treatment‐resistant depression [31]. Numerous clinical trials on omega‐3 fatty acids in depression have been published; a meta‐analysis of 53 studies found that omega‐3 supplementation significantly alleviated depressive symptoms [32]. Moreover, a network meta‐analysis of 10 trials comparing various dosages of omega‐3 supplementation demonstrated that both low and high doses significantly improved depressive symptoms compared to placebo, with high doses showing a more pronounced effect than low doses [33]. A meta‐analysis of 28 trials comparing EPA and DHA, two principal components of omega‐3 fatty acids, indicated that while DHA did not significantly alleviate depressive symptoms, EPA resulted in notable symptom improvement, highlighting its therapeutic potential [34]. Findings from the RCTs included in this review also indicated that EPA significantly improved depressive symptoms compared to placebo and DHA, consistent with broader evidence on depression. Thus, omega‐3 fatty acids, particularly EPA, could be considered a potential treatment for mild depression.

Other food‐derived compounds, such as magnesium, found in nuts, and SAMe, present in yeast, may also hold therapeutic potential for depression. Animal studies have shown that mice fed a low‐magnesium diet exhibited increased depressive‐like behavior, suggesting a causal relationship between magnesium deficiency and depression [35]. In a study involving 60 patients with depression and low magnesium levels, those who received 500 mg of magnesium experienced significantly lower scores on the BDI‐II compared to those who received a placebo [36]. In an RCT included in this review, magnesium chloride was effective for milder depression compared to a no‐treatment group, indicating its potential utility. Given their low invasiveness, obtaining these nutrients through dietary sources might be recommended as an initial treatment option. Conversely, while a meta‐analysis of 11 trials for general depression found that SAMe significantly improved depressive symptoms compared to placebo [37], no significant improvement was observed in the RCTs included in this review. This suggests further evidence is needed for SAMe to be considered a useful treatment option for mild depression.

St. John's Wort, recommended as a first‐line treatment for mild depression in the CANMAT guidelines, has strong evidence supporting its efficacy in depression [5]. A meta‐analysis of 27 trials found that St. John's Wort had comparable efficacy to SSRIs with a significantly lower dropout rate [38]. In an RCT included in the present review, a study by Moreno that reported negative findings on St. John's Wort showed a high risk of bias compared to other studies, suggesting that this study alone may not accurately represent its efficacy. In the present meta‐analysis, the inclusion of Moreno's study alongside Harrer's study demonstrated that the response rate for St. John's Wort was comparable to that of fluoxetine, further indicating its potential equivalence to antidepressants for mild depression. In both studies, St. John's Wort was associated with fewer adverse events than fluoxetine, and although the difference was not statistically significant, the dropout rate was approximately half that of fluoxetine. Consequently, St. John's Wort may be regarded as a viable treatment option, particularly in cases where maintaining treatment adherence is of paramount importance.

Other herbs, while less studied than St. John's Wort, have also shown promising efficacy. *Rhodiola rosea* significantly improved depressive symptoms compared to placebo in an RCT involving 89 participants [39]. Rhodiola, used as an adjunct therapy to sertraline in the study included in the present review, demonstrated significant improvement over placebo in treating milder depression, suggesting it might serve as a valuable complementary treatment. Lavender has shown efficacy in an RCT of 45 patients, where the addition of lavender to imipramine significantly improved depressive symptoms compared to imipramine alone [40]. An RCT of lemon balm involving diabetic patients with comorbid depression reported that 60 patients in the lemon balm group exhibited significant symptom improvement compared to placebo [41]. In the study included in the present review, lavender and lemon balm demonstrated effects comparable to antidepressants for treating milder depression. While no statistically significant differences were identified for lavender and lemon balm compared to fluoxetine, both were associated with a lower incidence of adverse events, including sexual dysfunction, appetite loss, and insomnia. TECAS was effective for treating mild depression and demonstrated a statistically significantly lower incidence of adverse events compared to fluoxetine, with the rate being less than half that observed with fluoxetine. A survey conducted in Japan indicated that 28.9% of individuals who discontinued their medication without consulting a physician attributed their decision to adverse events [42]. This underscores the potential for treatments with fewer adverse effects to enhance medication adherence. Accordingly, similar to St. John's Wort, these supplement therapies might be considered viable options in situations where maintaining treatment continuity is a priority.

This study has several limitations. First, the lack of standardization in diagnostic criteria across the included studies poses a significant challenge. Although diagnoses were predominantly based on DSM or ICD criteria, inconsistent application of these standards may have introduced discrepancies. Furthermore, depression severity was assessed using various scales, including the MADRS and HAM‐D, which complicates direct comparisons across studies and may contribute to inconsistencies in the reported outcomes. Second, the included trials exhibited substantial variability in duration and participant demographics. While some studies encompassed a broad age range, others predominantly focused on older populations, potentially limiting the generalizability of the findings to specific patient subgroups. Third, the literature search was confined to PubMed and Embase, potentially excluding relevant studies indexed in other databases, such as PsycINFO or Web of Science. This restriction raises concerns regarding the comprehensiveness of the evidence, particularly with respect to studies published in non‐English languages or in less commonly indexed journals. Fourth, the ROB2 assessment identified a high risk of bias in several studies, casting doubt on the reliability of certain included articles. Additionally, the studies incorporated in the present meta‐analysis displayed notable heterogeneity and a high risk of bias, further limiting the robustness of the findings. Furthermore, the potential limitations associated with publication bias, which may lead to an overestimation of the efficacy of supplements, warrant careful consideration. For example, analyses of publication bias in studies on omega‐3 fatty acids have indicated that positive findings may be influenced by such biases [43]. Finally, although this review sought to provide a thorough evaluation of pharmacological interventions for mild depression, the scarcity of high‐quality RCTs specifically targeting this population constrains the reliability and applicability of the conclusions.

In conclusion, this systematic review identified multiple studies on pharmacological interventions, primarily supplements, for milder depression. The search results also highlighted the lack of RCTs directly comparing antidepressants to placebo specifically in cases of mild depression. The findings of this systematic review do not provide sufficient evidence to support definitive clinical recommendations, underscoring the need for further research to evaluate the efficacy and safety of pharmacological interventions in mild depression.

## Ethics Statement

The authors have nothing to report.

## Consent

The authors have nothing to report.

## Conflicts of Interest

MU has received speaker honoraria from Sumitomo Pharma and Janssen Pharmaceutical over the last 3 years. HS received grants from the Japan Society for the Promotion of Science, the Japan Research Foundation Clinical Pharmacology, and the Takeda Science Foundation, and an honorarium from Viatris, Eisai, Takeda Pharmaceutical, Otsuka Pharmaceutical, Meiji Seika Pharma, Shionogi Pharma, Yoshitomiyakuhin, Sumitomo Pharma, Kyowa Pharmaceutical, MSD, and Lundbeck Japan. HS is an Editorial Board member of Neuropsychopharmacology Reports and a corresponding author of this article. To minimize bias, they were excluded from all editorial decision‐making related to the acceptance of this article for publication. FU has received grants from the Nakatani Foundation, the Canadian Institutes of Health Research (CIHR), and the Brain & Behavior Research Foundation (BBRF); manuscript fees from Dainippon Sumitomo Pharma; and consultant fees from WCG Clinical and Uchiyama Underwriting within the past three years. TM has nothing to declare. T. Tada has received speaker honoraria from Dainippon Sumitomo Pharma and Otsuka Pharmaceutical. TU has nothing to declare. YM received an honorarium from Sumitomo Pharma, Janssen Pharmaceutical, and Meiji Seika Pharma. MM received an honorarium from Sumitomo Pharma, Yoshitomiyakuhin. MT has nothing to declare. HB received grant funding from the Japan Society for the Promotion of Science and speaker's honoraria from Otsuka Pharmaceutical, Sumitomo Dainippon Pharma, Viatris, MSD, Meiji Seika Pharma, Eli Lilly, Yoshitomi Yakuhin, Janssen Pharmaceutical, Kyowa Pharmaceutical, Mitsubishi Tanabe Pharma, Pfizer, Esai, Takeda Pharmaceutical, Lundbeck Japan, Mochida, Sawai, Kowa, EA Pharma, and Mylan EPD. MK has received grant funding from AMED, the Japanese Ministry of Health, Labour and Welfare, the Japan Society for the Promotion of Science, SENSHIN Medical Research Foundation, the Japan Research Foundation for Clinical Pharmacology, and the Japanese Society of Clinical Neuropsychopharmacology, and consulting fees from Sumitomo Pharma Co. Ltd., Shionogi & Co. Ltd., Otsuka Pharmaceutical Co. Ltd., Lundbeck Japan K.K., Boehringer Ingelheim Co. Ltd., and Takeda Pharmaceutical Co. Ltd.; payment or honoraria for lectures, presentations, speakers' bureaus, manuscript writing, or educational events from Sumitomo Pharma Co. Ltd., Otsuka Pharmaceutical Co. Ltd., Meiji Seika Pharma Co. Ltd., Shionogi & Co. Ltd., Mitsubishi Tanabe Pharma Corporation, Takeda Pharmaceutical Co. Ltd., Lundbeck Japan K.K., Viatris Inc., Eisai Co. Ltd., and Kyowa Pharmaceutical Industry Co. Ltd. T. Tsuboi received grants from the Japan Society for the Promotion of Science and an honorarium from Takeda Pharmaceutical, Otsuka Pharmaceutical, Meiji Seika Pharma, Shionogi Pharma, Yoshitomiyakuhin, Sumitomo Pharma, Kyowa Pharmaceutical, MSD, Nippon Boehringer Ingelheim, Mylan EPD, Mitsubishi Tanabe Pharma, Viatris, Mochida Pharmaceutical, Janssen Pharmaceutical, TEIJIN PHARMA, and Lundbeck Japan. KW has received consultant fees from Boehringer Ingelheim, Daiichi Sankyo, Eisai, Lundbeck Japan, Luye Pharma, Mitsubishi Tanabe Pharma, Nippon Chemiphar, Ono Pharmaceutical, Otsuka Pharmaceutical, Sumitomo Pharma, and Takeda Pharmaceutical, received grant funding from AMED, the Japan Society for the Promotion of Science, and speaker honoraria from Boehringer Ingelheim, Eisai, Janssen Pharmaceutical, Kyowa Pharmaceutical, Lundbeck Japan, Meiji Seika Pharma, Mitsubishi Tanabe Pharma, MSD, Otsuka Pharmaceutical, Shionogi, Sumitomo Pharma, Takeda Pharmaceutical, and Viatris.

## Registry and the Registration No. of the Study/Trial

Open Science Framework (https://osf.io/7a3fe).

## Permission to Reproduce Material From Other Sources

We own all the rights in the material submitted and agree to transfer, assign, or otherwise convey all copyright ownership to the Neuropsychopharmacology Reports upon acceptance of this manuscript.

## Supporting information


**Table S1.** Search strategies.

## Data Availability

The articles cited in the present paper are openly available in PubMed or Embase.
