# Supplementary material for: Efficacy of Pharmacological Interventions in Milder Depression: A Systematic Review and Meta‐Analysis
Source: Neuropsychopharmacol Rep. 2025 Feb 27;45(1):e70008. doi: 10.1002/npr2.70008 (PMC11867163; doi:10.1002/npr2.70008)
Supplement: Supplementary file 1 — Table S1. Search strategies. [file NPR2-45-e70008-s001.docx]

**Supplementary information**

**Table S1. Search strategies**

We searched the databases of PubMed and Embase using the following term:

PubMed

(mild*[Title/Abstract] OR minor*[Title/Abstract] OR subthreshold*[Title/Abstract]) AND depress*[Title/Abstract] AND ("Antidepressive Agents"[Mesh] OR antidepressant*[Title/Abstract] OR selective serotonin reuptake inhibitor*[Title/Abstract] OR SSRI[Title/Abstract] OR SSRIs[Title/Abstract] OR fluoxetine[Title/Abstract] OR fluvoxamine[Title/Abstract] OR paroxetine[Title/Abstract] OR sertraline[Title/Abstract] OR citalopram[Title/Abstract] OR escitalopram[Title/Abstract] OR vortioxetine[Title/Abstract] OR serotonin norepinephrine reuptake inhibitor*[Title/Abstract] OR SNRI[Title/Abstract] OR SNRIs[Title/Abstract] OR duloxetine[Title/Abstract] OR venlafaxine[Title/Abstract] OR desvenlafaxine[Title/Abstract] OR milnacipran[Title/Abstract] OR levomilnacipran[Title/Abstract] OR mianserin[Title/Abstract] OR nefazodone[Title/Abstract] OR trazodone[Title/Abstract] OR vilazodone[Title/Abstract] OR bupropion[Title/Abstract] OR reboxetine[Title/Abstract] OR agomelatine[Title/Abstract] OR noradrenergic and specific serotonergic antidepressant*[Title/Abstract] OR NaSSA[Title/Abstract] OR NaSSAs[Title/Abstract] OR mirtazapine[Title/Abstract] OR TCA[Title/Abstract] OR TCAs[Title/Abstract] OR tricyclic[Title/Abstract] OR amersergide[Title/Abstract] OR amineptine[Title/Abstract] OR amitriptyline[Title/Abstract] OR amoxapine[Title/Abstract] OR butriptyline[Title/Abstract] OR chlorpoxiten[Title/Abstract] OR clomipramine[Title/Abstract] OR clorimipramine[Title/Abstract] OR demexiptiline[Title/Abstract] OR desipramine[Title/Abstract] OR dibenzipin[Title/Abstract] OR dothiepin[Title/Abstract] OR doxepin[Title/Abstract] OR imipramine[Title/Abstract] OR lofepramine[Title/Abstract] OR melitracen[Title/Abstract] OR metapramine[Title/Abstract] OR nortriptyline[Title/Abstract] OR noxiptiline[Title/Abstract] OR opipramol[Title/Abstract] OR protriptyline[Title/Abstract] OR quinupramine[Title/Abstract] OR tianeptine[Title/Abstract] OR trimipramine[Title/Abstract] OR sulpiride[Title/Abstract] OR buspirone[Title/Abstract] OR aripiprazole[Title/Abstract] OR brexpiprazole[Title/Abstract] OR serotonin dopamine activity modulator*[Title/Abstract] OR SDAM[Title/Abstract] OR SDAMs[Title/Abstract] OR partial dopamine agonist*[Title/Abstract] OR olanzapine[Title/Abstract] OR quetiapine[Title/Abstract] OR multi acting receptor targeted antipsychotic*[Title/Abstract] OR MARTA[Title/Abstract] OR MARTAs[Title/Abstract] OR ketamine[Title/Abstract] OR esketamine[Title/Abstract] OR arketamine[Title/Abstract] OR N methyl D aspartate antagonist*[Title/Abstract] OR NMDA antagonist*[Title/Abstract])

Embase

((mild* or minor* or subthreshold*) and depress* and ('Antidepressive Agents' or 'antidepressant' or 'selective serotonin reuptake inhibitor' or 'SSRI' or 'SSRIs' or 'fluoxetine' or 'fluvoxamine' or 'paroxetine' or 'sertraline' or 'citalopram' or 'escitalopram' or 'vortioxetine' or 'serotonin norepinephrine reuptake inhibitor' or 'SNRI' or 'SNRIs' or 'duloxetine' or 'venlafaxine' or 'desvenlafaxine' or 'milnacipran' or 'levomilnacipran' or 'mianserin' or 'nefazodone' or 'trazodone' or 'vilazodone' or 'bupropion' or 'reboxetine' or 'agomelatine' or 'noradrenergic and specific serotonergic antidepressant' or 'NaSSA' or 'NaSSAs' or 'mirtazapine' or 'TCA' or 'TCAs' or 'tricyclic' or 'amersergide' or 'amineptine' or 'amitriptyline' or 'amoxapine' or 'butriptyline' or 'chlorpoxiten' or 'clomipramine' or 'clorimipramine' or 'demexiptiline' or 'desipramine' or 'dibenzipin' or 'dothiepin' or 'doxepin' or 'imipramine' or 'lofepramine' or 'melitracen' or 'metapramine' or 'nortriptyline' or 'noxiptiline' or 'opipramol' or 'protriptyline' or 'quinupramine' or 'tianeptine' or 'trimipramine' or 'sulpiride' or 'buspirone' or 'aripiprazole' or 'brexpiprazole' or 'serotonin dopamine activity modulator' or 'SDAM' or 'SDAMs' or 'partial dopamine agonist' or 'olanzapine' or 'quetiapine' or 'multi acting receptor targeted antipsychotic' or 'MARTA' or 'MARTAs' or 'ketamine' or 'esketamine' or 'arketamine' or 'N methyl D aspartate antagonist' or 'NMDA antagonist')).ab,ti.
